# Femoral Head Fracture Fixation Using Femoral Head Cement Templating for Iliac Bone Graft and Biodegradable Screws via Ganz Surgical Dislocation of the Hip: A Case Report

**DOI:** 10.7759/cureus.70429

**Published:** 2024-09-29

**Authors:** Muhammad Ikmal Hazli, Abdul Qayyum Mohd Raziff, Zamri Ab Rahman

**Affiliations:** 1 Orthopedics and Traumatology, Hospital Universiti Kebangsaan Malaysia, Cheras, MYS; 2 Orthopedics, Hospital Tuanku Ja'afar Seremban, Negeri Sembilan, MYS

**Keywords:** bioabsorbable screw, bone cement templating, femoral head fracture, ganz osteotomy, iliac bone graft, pipkin fracture

## Abstract

A 22-year-old Malaysian male presented with a closed posterior dislocation of the right hip with femoral head fracture (Pipkin II) after an alleged motor vehicle accident (MVA). This type of injury may pose future complications, such as avascular necrosis, post-traumatic osteoarthritis, or heterotopic ossification to the hip joint, and later affect joint function. After an immediate closed reduction of the hip, the patient underwent Ganz Surgical dislocation of the hip - a surgical method used to aid the reconstruction of the bony defect of the femoral head. Using bone cement templating, right iliac bone graft was harvested to support the bony defect of the femoral head, and biodegradable screws were inserted to reconstruct the femoral head. The use of absorbable screws eliminated the need for a second surgery to remove hardware, reducing complications.

At eight months post-operatively, the patient was able to ambulate without walking aid and fully bear weight on his right hip without pain. Imaging confirmed proper alignment and healing, with no indication for further surgery. This favorable outcome highlighted the efficacy of biodegradable screws in maintaining femoral head integrity while minimizing future surgical intervention.

One year later, the patient regained excellent functional capacity, achieving a Lower Extremity Functional Scale (LEFS) score of 96.3%, indicating near-complete recovery with minimal limitations.

This case report illustrates the success of using cement-templated iliac bone graft combined with biodegradable screws in femoral head fixation. The approach not only achieved structural stability but also led to a high functional outcome without the need for additional procedures. These findings support the use of absorbable fixation in complex orthopedic cases such as hip dislocations with femoral head involvement.

## Introduction

Femoral head fractures are relatively uncommon, occurring in 1-16.8% of patients with traumatic hip dislocation [1]. Pipkin classified these fractures into four types [2]. While there have been occasional reports of conservative management for reducible and non-displaced Pipkin fractures, [3,4] the general consensus is to surgically stabilize displaced fractures as soon as possible [1]. Some authors advocate for the removal of smaller femoral head fragments, while others prefer fixation using intra-articular or extra-articular Herbert screws or small fragment screws. However, using these conventional methods, only 30% of patients with Pipkin type I and type II fractures and 50% of those with type III and type IV fractures achieve outcomes that are moderate or worse [3].

In terms of complications, approximately 14.5% of patients with Pipkin type I and type II fractures develop femoral head avascular necrosis (AVN), while 15.4% experience hip arthritis and periarticular ossification [3]. For those with Pipkin Type III and IV fractures, 50% develop arthritis and femoral head necrosis and 80% develop periarticular sclerosis [1,3,5].

A potential explanation for these suboptimal outcomes (with satisfactory outcomes defined as painless full weight-bearing and good range of motion) after internal fixation with screws is the protrusion of the screw head or tip into the joint, leading to articular abrasion and subsequent joint damage. When placing screws extra-articularly, it can be difficult to secure small fracture fragments effectively, as only a few threads of the screw engage the fragment before the tip protrudes through the cartilage. For intra-articularly placed screws, screw head protrusion can be avoided by countersinking the head below the cartilage surface. However, if removal of the hardware becomes necessary, intra-articular screws require re-dislocation of the hip for removal.

A biodegradable implant may offer a solution to these challenges, as it can be placed intra-articularly with the head countersunk below the cartilage, while also being bioresorbable, eliminating the need for a second procedure for removal. In this case, the screws used for fixation of the right femoral head defect were absorbable screws made from a poly-L-lactic acid + hydroxyapatite composite.

## Case presentation

**Figure 1:**
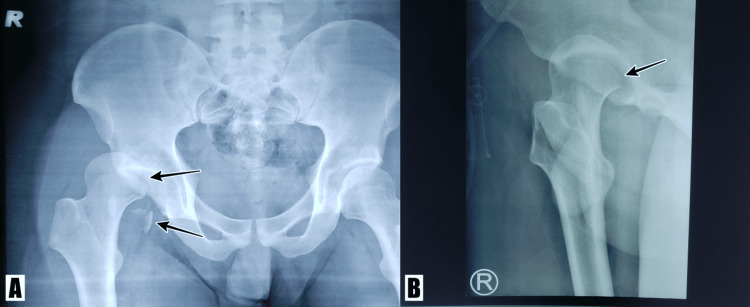
Initial plain radiographs post-trauma (A) Plain radiograph of the pelvis (anteroposterior view) and (B) right hip (lateral view) showing posterior dislocation of the right hip with femoral head fracture (arrows).

A 22-year-old Malaysian male presented after an alleged motor vehicle accident (MVA) in December 2021. The patient was riding a motorcycle when an oncoming car suddenly swerved into his lane, and as he tried to avoid collision with the oncoming car, he lost control of his motorcycle and hit the divider over his right thigh. Post-trauma, the patient was unable to ambulate. An ambulance arrived at the scene, the patient was put on a lower limb splint and brought to our hospital. In the emergency department (ED), his vitals were stable, and clinical examination revealed that the patient had relative shortening of the right lower limb compared to the left, and his hip was in flexion and adducted, with internal rotation seen. There were no neurological deficits, and the pulses were all present. Plain radiographs revealed that the patient had sustained right posterior hip dislocation with femoral head fracture (Figure 1).

Under sedation, multiple attempts of closed manipulative reduction (CMR) were made in the ED to reduce the dislocated right hip, but it was not successful, and subsequently a supracondylar pin of the right femur was inserted for traction of the right lower limb with Bohler Braun frame, and 9kg of weight traction was applied as a means of immobilization. After failed CMR and supracondylar pin insertion, the patient did not have foot drop or loss of sensation and was able to move his ankle and toes. The distal pulses (dorsalis pedis artery and posterior tibial artery) were also palpable.

**Figure 2:**
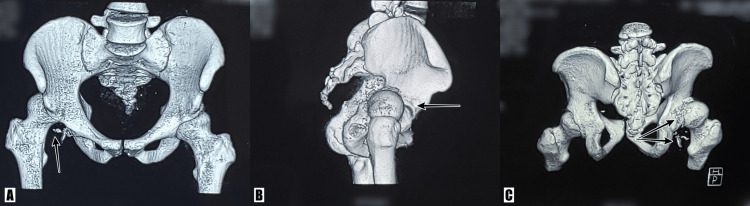
Computed tomography (3D reconstruction) of the pelvis (A, B) A 3D CT reconstruction view showing posterior dislocation of the right hip (arrows). (C) A 3D CT reconstruction view showing right femoral head fracture with multiple fragments (arrows). 3D, three-dimensional; CT, computed tomography

A CT scan of the hip (Figure 2) was performed, which revealed posterior dislocation of the right femoral head, which was in contact with the posterior rim of the acetabulum with depressed fracture at its posteromedial aspect and the posterior acetabular rim. Multiple comminuted bony fragments were seen inferior and posteriorly, along with small intra-articular bone fragments. The CT scan aided us in classifying the fracture pattern (Pipkin II) and assisted our pre-operation planning.

The patient was planned for operation because of the failed reduction of the hip and to address the femoral head fracture. The planned surgery for the patient was for re-CMR of the right hip, with reconstruction of the femoral head using iliac bone graft with cement templating and bioabsorbable screw insertion using Ganz surgical dislocation of the hip. During the operation, the right femur was anteriorly dislocated to increase the direct surgical view field of the whole femoral head. Intra-operative findings showed fracture over the right femoral head involving the supra fovea and infra fovea measuring 6cm x 3cm, as well as comminuted small fragments of the femoral head that were unconstructable.

**Figure 3:**
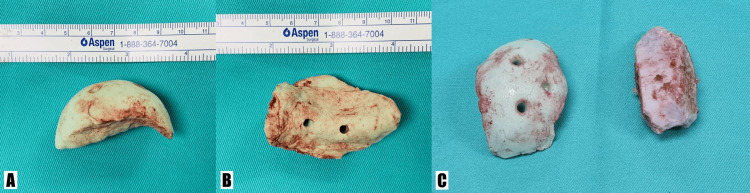
Bone cement templating and harvested iliac bone graft (A, B) Bone cement templating of the femoral head defect measuring approximately 6x3cm. (C) Side-by-side comparison of the bone cement template (left) with the harvested iliac bone graft (right).

In this case, bone cement was used as a template to serve as a gauge to approximate the volume of iliac bone graft that would be needed to fill the defect onto the bone loss over the femoral head (Figure 3).

**Figure 4:**
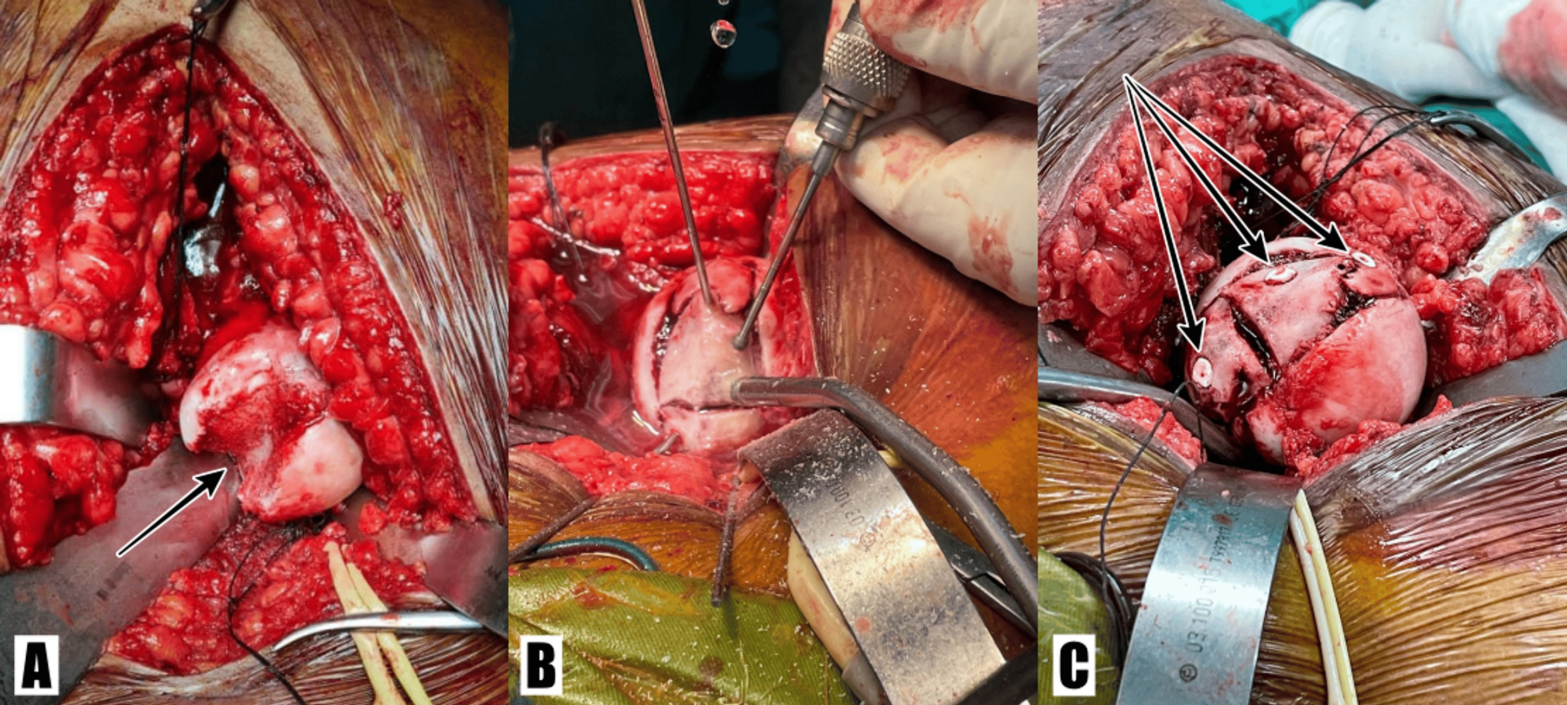
Intra-operative images of femoral bone defect and bone graft fixation (A) "Pacman" bone defect over femoral head (arrow). (B) K-wires were used to hold bone graft pieces in place. (C) Bioabsorbable screws were inserted onto the femoral head (arrows).

Bone graft from the right iliac bone were broken into parts to follow the contour of the femoral head, inserted at the femoral impaction site, and held with positional K-wires. Then the bone graft parts were fixed into place using three 3.5mm cannulated (absorbable) poly-L-lactic acid + hydroxyapatite screws (Figure 4). Coincident intra-operative findings revealed partial tear over the superior and inferior labrum, minimal fracture of posterior wall brim, and capsulolabrum junction tear. The trochanter (osteotomized via Ganz trochanteric osteotomy) was then fixed with two cancellous (one partial threaded + one half treaded) 7.0mm screws.

Post-operatively, an abduction pillow was applied to prevent adduction of the right lower limb. Skin traction (5kg weight) was then applied for two weeks in the ward, and the patient was restricted from any form of weight-bearing for three months. During the four-month post-operative check-up in clinic, the patient was able to weight bear and ambulate without any crutches or walking aid assistance, and was referred to our rehab team for right lower limb range-of-motion exercises. During the five-month post-operative check-up, the patient had better range of motion (full range of motion) over the right hip and was able to fully squat down. The patient achieved an excellent Lower Extremity Functional Scale (LEFS) score of 96.3% at one-year post-operative follow-up.

**Figure 5:**
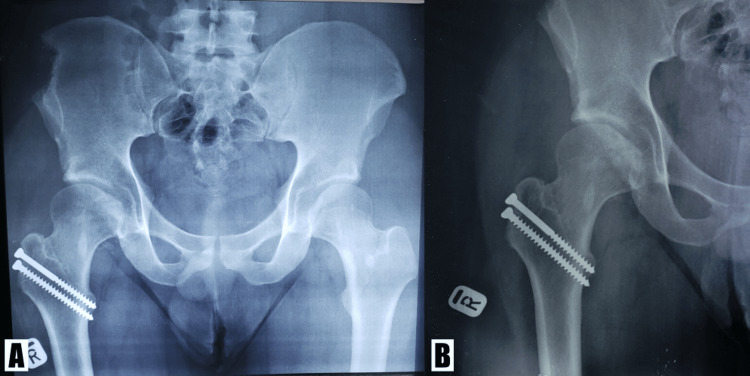
One-year post-operative plain radiographs (A) X-ray of the pelvis (anteroposterior view) and (B) right hip showing good bone union and shape of the femoral head, with no radiological evidence of AVN. AVN, avascular necrosis

X-rays at one year post-operatively showed good incorporated iliac graft over the right femoral head. It was also noted that the femoral head shape was well maintained, with no radiographical evidence of AVN on the right femoral head (Figure 5).

## Discussion

In this particular case, the patient was a young, physically active male, employed as an assistant technician. His job requires a significant degree of physical mobility, and maintaining his functional independence was essential to ensure both his quality of life and his ability to perform daily activities. Therefore, one of the primary objectives in managing this case was to preserve the integrity of the affected hip joint for as long as possible. Fixation of the right femoral head was chosen as a means to achieve this goal, given that the patient’s youth and active lifestyle made joint preservation particularly important.

AVN of the femoral head is a condition closely associated with impaired vascularization of the femoral head, which leads to progressive ischemia of the bone tissue. In the absence of an adequate blood supply, bone necrosis ensues, resulting in structural collapse of the bone over time. In this specific case, the underlying risk of AVN was trauma (posterior dislocation of the right hip, with femoral head fracture). Traumatic events such as these are well-documented in the literature as significant risk factors for the development of AVN due to the disruption of the delicate blood vessels supplying the femoral head [6].

AVN of the femoral head is marked by apoptosis of key bone cells, including those involved in bone formation (osteoblasts), bone destruction (osteoclasts), and bone marrow cells. The loss of these cells ultimately leads to the structural weakening of the bone, resulting in its collapse. As the disease progresses, the collapse of the bony structure eventually extends to the overlying articular cartilage, which plays a critical role in maintaining smooth, pain-free joint function. This cascade of events causes flattening of the head surface of the femur, which can lead to altered biomechanics of the hip joint. Over time, this altered joint anatomy results in the gradual development of secondary osteoarthritis, which further exacerbates the loss of joint function and can lead to chronic pain and disability [7].

In this patient, the surgical intervention involved the use of absorbable poly-L-lactic acid combined with hydroxyapatite screws. The advantage of using these absorbable materials is that they do not require a second operation for removal, as would be necessary with traditional metallic screws. This choice of fixation material not only simplifies the post-operative care process but also reduces the overall number of surgical procedures the patient must undergo, thus minimizing the risks associated with multiple surgeries, such as infection, anesthesia-related complications, and additional recovery time. By opting for this technique, we aimed to provide a durable solution that would preserve the joint for as long as possible.

Despite the initial success of the fixation, it is important to recognize that this patient may still be at risk of developing long-term complications, such as AVN or post-traumatic arthritis, as a direct consequence of the trauma he sustained. These conditions may manifest gradually, over the course of years, as the degenerative changes continue to affect the hip joint. However, the decision to proceed with fixation of the right femoral head was primarily driven by the desire to delay the onset of these complications and to maximize the period of functional use of the joint in this young patient. Early intervention is crucial in young, active individuals, as it can help maintain mobility and reduce the impact of joint degeneration on their overall lifestyle.

At the one-year follow-up post-operatively, the patient reported a highly favorable functional outcome, with an LEFS score of 96.3%. This score is indicative of a near-optimal functional status, suggesting that the patient experienced minimal limitations in activities of daily living and work-related tasks. The high LEFS score also reflects the success of the surgical intervention in restoring functional capacity and mobility in this patient. Nevertheless, it is important to closely monitor the patient for potential late-onset complications. Should AVN or hip arthritis develop over time, further surgical intervention, such as arthroplasty, may be necessary to address the resultant joint degeneration. Arthroplasty, particularly total hip replacement, could be considered in the future if conservative management options fail to adequately manage symptoms or if the joint becomes severely compromised.

## Conclusions

Femoral head fractures are usually associated with high-energy collisions that cause hip dislocation, and urgent reduction is needed to prevent complications such as AVN of the femoral head. Fixation or reconstruction of the femoral head serves a purpose to delay the onset of these complications and can especially benefit younger patients to prolong or delay the need for arthroplasty surgeries for the patient. In this case, the use of femoral head cement templating ensured that the volume of harvested iliac bone was adequate for the femoral head reconstruction, and the choice of using (bioabsorbable) poly-L-lactic acid + hydroxyapatite screws avoided the need for subsequent operations as they do not require removal of implant. These combined techniques helped in achieving a stable hip with good functional outcome for the patient.
